# Diagnosis, Characteristics, and Outcome of Selective Anti-polysaccharide Antibody Deficiencies In A Retrospective Cohort of 55 Adult Patients

**DOI:** 10.1007/s10875-025-01874-2

**Published:** 2025-03-17

**Authors:** Nicolas Perrard, Sarah Stabler, Sébastien Sanges, Louis Terriou, Catherine Lamblin, Sacha Gaillard, Fanny Vuotto, Cécile Chenivesse, Geoffrey Mortuaire, Frédéric Batteux, Floriane Mirgot, Aurore Collet, Benjamin Lopez, Sylvain Dubucquoi, Myriam Labalette, Eric Hachulla, David Launay, Guillaume Lefèvre

**Affiliations:** 1 Department of Internal Medicine and Clinical Immunology, CHU Lille, F-59000 Lille, France; 2 University of Lille, Inserm, CHU Lille, U1286 - INFINITE - Institute for Translational Research in Inflammation, F-59000 Lille, France; 3 Institute of Immunology, CHU Lille, F-59000 Lille, France; 4 Regional Center for Primary Immune Deficiency, CEREDIH Lille, CHU Lille, F-59000 Lille, France; 5 Department of Infectious and Tropical Diseases, CHU Lille, F-59000 Lille, France; 6 U1019 - UMR 9017 - CIIL - Center for Infection and Immunity of Lille, University of Lille, CNRS, Inserm, CHU Lille, Institut Pasteur de Lille, F-59000 Lille, France; 7 Department of Pneumology, Private Hospital La Louvière, F-59000 Lille, France; 8 Department of Pneumology, Clinique Tessier, F-59300 Valenciennes, France; 9 Department of Pneumology, CHU Lille, F-59000 Lille, France; 10 Department of Oto-Rhino-Laryngology, CHU Lille, F-59000 Lille, France; 11 Department of Biological Immunology, Cochin Hospital, F-75014 Paris, France; 12 Cochin Institute, INSERM U1016, University of Paris, F-75014 Paris, France; 13 CH Dunkerque, Medical Laboratory Department, F-59240, Dunkirk, France

**Keywords:** Specific antibody deficiency, Selective anti-polysaccharide antibody deficiency, Primary immunodeficiency, Encapsulated bacterial infections, Preventive antibiotherapy, Immunoglobulin replacement therapy, Asthma

## Abstract

Selective anti-polysaccharide antibody deficiency (SPAD) predisposes to encapsulated bacterial infections. The diagnosis is challenging, and literature reports are scarce in adult patients, we therefore aim to describe the demographics, infectious complications, therapeutic strategies, and outcome of adult patients. We conducted a multicenter observational study involving 55 adult patients with SPAD. The median [interquartile range, IQR] age was 45 [36–60] years at diagnosis of SPAD, and 75% of patients were female. Twenty-one patients (38%) had a history of allergic and/or inflammatory disease, mainly asthma (*n* = 12), and rheumatic diseases (*n* = 6). Twelve patients (22%) were diagnosed after a single severe infection and 43 (78%) in a context of recurrent benign and/or severe infections. In the latter, the median time from first infections to diagnosis was 74.5 [33–167] months. Diagnostic delay was significantly higher in patients presenting with bronchiectasis than in those without (122 months [33–219.5] vs 24 months [14.5–74.5], *p* = 0.0042). In 22 patients (40%) receiving immunoglobulin replacement therapy (IgRT), the mean (min–max) frequency of antibiotic courses decreased from 7.9 (2–18) to 0.7 (0–2) courses per year (*p* < 0.001) with a median follow-up period of 46 [27–73] months. Patients diagnosed after a single severe infection did not have any relapse during a median follow-up of 85 [80.5–104.5] months after diagnosis. Adult patients with SPAD have allergic or inflammatory disorders which could contribute to the diagnostic delay. IgRT is effective in preventing recurrent infections. Further studies are warranted to confirm if SPAD should be considered after a first unexplained severe bacterial infection.

**Supplementary Information:**

The online version contains supplementary material available at 10.1007/s10875-025-01874-2.

## Introduction

Primary immunodeficiencies (PIDs) encompass rare disorders predisposing individuals to various infections, autoimmune diseases, and neoplasia [1]. Antibody deficiencies typically manifest through bacterial upper and/or lower respiratory tract infections (U/LRTIs), with pathogens such as *Streptococcus pneumoniae*, *Streptococcus pyogenes*, and *Haemophilus influenzae* [2, 3]. Among antibody deficiencies, the first fully characterized case of specific anti-polysaccharide antibody deficiency (SPAD) was reported in 1987: Ambrosino and colleagues described an adult patient with a history of recurrent U/LRTIs, demonstrating impaired antibody responses to polysaccharide (PS) antigens but normal responses to protein antigens [4]. SPAD is now characterized by an impaired response to PS antigens, despite normal response to protein antigens, and normal levels of immunoglobulin (Ig), IgG subclasses, T cells, and B cells [5, 6]. This assessment of anti-PS response is made by evaluating antibodies against pneumococcal capsular polysaccharides (PnPS), using a standardized, serotype-by-serotype approach before and 4 to 8 weeks after immunization with the non-conjugate 23-valent polysaccharide pneumococcal vaccine (PPV23, PNEUMOVAX®) [7].

In a recent multicenter prospective study, we demonstrated that SPAD may represent a common etiology for recurrent benign and/or severe bacterial infections when local or systemic predisposing conditions are excluded [8]. However, several considerations remain unknown in the diagnosis and management of SPAD in adult patients. First, clinical practice shows that some SPAD patients could have common chronic respiratory diseases, tobacco use, or inflammatory disorders, even if these could be considered factors predisposing to infections [9]. Second, the benefit of anti-PS response assessment in patients who have had a single unexplained severe infection needs to be confirmed [8]. Third, the long-term benefit of SPAD diagnosis and the efficacy of IgRT require further evaluation.

In this study, we aimed to describe the demographics, diagnosis, infectious complications, immunological characteristics, therapeutic strategies, and outcome of adult patients with SPAD.

## Methods

### Patients

SPAD diagnosis was made if a patient had unexplained recurrent bacterial infections or at least one severe infection with encapsulated bacteria, normal levels of Ig and IgG subclasses, normal B cell counts, and an impaired anti-polysaccharide response 4 to 8 weeks after vaccination with PPV23. The absence of vaccination with PPV23 at least two years prior to testing was also required to avoid the hypo-responsiveness phenomenon. Interpretation of anti-PnPS response and SPAD diagnosis were defined in accordance with the recommendations of the American Academy of Allergy, Asthma & Immunology (AAAAI) [2, 7], and assessing pre- and postimmunization anti-PnPS antibody titers using the third-generation World Health Organization enzyme-linked immunosorbent serotype-specific assay (SSA) [2, 7, 10]. An insufficient response for a given serotype was considered to correspond to post-immunization antibody titers below 1.3 mg/L or failing to show a fourfold increase. A twofold increase was deemed acceptable if the initial value was already greater than 1.3 mg/L. A poor response to PnPSs was defined as an insufficient response to at least 70% of the tested serotypes (7 to 13), and patients were therefore diagnosed with SPAD. The diagnosis of SPAD was also established if the anti-PnPS antibodies remained below 110 mg/L using the overall anti-pneumococcal response assay (OVA), with a positive predictive value of 100% according to a previous work published by our team [11].

The degree of vaccine response impairment in selective antibody deficiency was defined according to the SSA, as follows: a mild phenotype when patients had either multiple vaccine-containing serotypes to which they did not generate protective titers ≥ 1.3 mg/mL or an inability to increase titers two-fold for ≥ 70% of serotypes, assuming the prevaccination titers are below the threshold levels described earlier; a moderate phenotype when patients had fewer than the expected number of protective titers for 70% of serotypes but demonstrated protective titers ≥ 1.3 mg/mL for three or more serotypes; and a severe phenotype when patients had protective titers for no more than two serotypes, with any titers present tending to be < 1.3 mg/mL [7].

CD27 + IgD-IgM- switched memory B cells were considered low if their count was below 6.5% of CD19 + B cells, and CD27 + IgD + IgM + marginal zone-like B cells were considered low if below 7.4% of total CD19 + B cell [12].

### Data Collection

Details of patients’ demographic characteristics and personal medical histories, encompassing autoimmune or inflammatory disorders, practitioner reports of asthma or allergies, neoplasia, and the presence of bronchiectasis, were documented from medical records. Biological parameters, including T-cell, B-cell (with naive/memory subsets), and NK-cell counts, and levels of IgG, IgA, and IgM, were recorded at the time of diagnosis and throughout follow-up when available.

Bacterial infections evocative of SPAD corresponded to documented infections with encapsulated bacteria, or, in the absence of documentation, to infections involving sites commonly affected by encapsulated bacteria, i.e., the pharynx and ENT region (URTI), the bronchi, and lungs (LRTI). There were defined as benign if treated in an outpatient setting, severe if necessitating hospitalization and/or intravenous antibiotics and recurrent if occurring at least twice per year for a minimum of two years). Patients where then classified in 3 infectious phenotypes Antibody responses to 13-valent conjugate polysaccharide vaccines (PCV13, PREVENAR 13®), and conjugate anti-*Haemophilus influenzae* serotype B HibCV, ACT-HIB®) were collected if at least one assessment was available within six months following immunization. Additional data were gathered on patients undergoing IgRT, including the frequency of infections and courses of antibiotics per year before and after IgRT initiation (at the latest follow-up visit). PCV13 was administered at least 1 year after the pneumococcal polysaccharide vaccine administered for diagnostic to avoid the hypo-responsiveness phenomenon.

### Statistical Analysis

Continuous variables are expressed as median (interquartile range [IQR]) and categorical variables are expressed as number (percentage).

Inter-group comparisons with regard to clinical and biological characteristics were performed using Student’s t test for continuous variables, and the chi-squared test or Fisher’s exact test for categorical variables, as appropriate.

Comparisons of infection frequency after IgRT versus baseline were done using the Wilcoxon’ signed rank test.

Data analyses and graphs were performed using the GraphPad Prism version 9.1.2 (GraphPad Software, La Jolla, CA).

All tests were two-tailed, and the threshold for statistical significance was set to *p* < 0.05.

### Ethics Statement

All patients were aged 18 or older, and non-opposition to data use was collected (“Protection des Données – GHT Lille Métropole Flandre intérieure”, Agreement No. DEC23-109). Data from the 36 patients included from the prospective multicenter study were collected after written consent from each patient, with the approval of our Institutional Review Board and by an independent ethics committee in accordance with French legislation (“Comité de Protection des Personnes Nord Ouest IV”, Agreement No. 2014–100739-38).

## Results

### Bacterial Infections and Immunological Characteristics

Fifty-five patients were included in the study (41 females [75%]), and the median (IQR) age at diagnosis was 45 years (36–60 years) (Table 1). Thirty-nine patients were diagnosed using a 7 serotype SSA and 10 using a 13 serotype SSA, the 6 remaining patients were diagnosed using the OVA [7, 11].

**Table 1:** Patients’ characteristics according to their clinical infectious phenotype

	SPAD Cohort (*n* = 55)	Single severe infection (*n* = 12)	Recurrent benign infections (*n* = 24)	Recurrent infections with ≥ 1 severe infection (*n* = 19)	*p*
Female gender, *n* (%)	41 (75)	8 (72)	18 (75)	15 (79)	*0.463*
Age (y) at diagnosis, median (IQR)	45 (36–60)	45.5 (36.5–67.5)	45.5 (40.5–52.5)	45 (35–61.5)	*0.738*
Infectious phenotype					
Single severe infection, *n* (%)	12 (22)				
Recurrent benign infections, *n* (%)	24 (44)				
Recurrent infections with ≥ 1 severe infection, *n* (%)	19 (34)				
Vaccine response impairment according to SSA^$^					
Mild, *n* (%)	18 (33)	5 (42)	9 (37)	4 (21)	*0.231*
Moderate, *n* (%)	12 (22)	2 (16)	4 (16)	6 (32)	*0.260*
Severe, *n* (%)	23 (42	5 (42)	9 (37)	9 (47)	*0.526*
Bacteriological documentation^*^, *n* (%)	24 (44)	12 (100)	3 (12)	9 (47)	< *0.001*
* Streptococcus pneumonia*, *n*	12	6	-	5	
* Neisseria meningitidis*, *n*	8	6	-	2	
* Haemophilus influenzae*, *n*	7	-	3	4	
* Streptococcus pyogenes*, *n*	2	-	1	*-*	
No documentation, *n*	31	0	21	10	
Bronchiectasis, *n* (%)	13 (24)	0 (0)	6 (25)	7 (37)	*0.016*
Allergic/Inflammatory diseases, *n* (%)	21 (38)	4 (33)	11 (58)	9 (47)	*0.457*
Asthma, *n*	12	1	7	4	
Inflammatory rheumatism^**^, *n*	6	2	1	3	
Autoimmune thyroiditis, *n*	4	0	2	2	
Sjögren's syndrome, *n*	2	0	0	2	
Psoriasis, *n*	2	1	0	1	
Auto-inflammatory disease, *n*	1	0	1	0	
Inflammatory bowel disease, *n*	1	0	1	0	
Biological only autoimmunity^***^, *n*	4	0	1	3	
Biological parameters (*n* = 48)					
Decreased NK cell, *n* (%)	12 (22)	3 (25)	5 (21)	4 (21)	*0.806*
Decreased switched memory B cells, *n* (%)	7 (13)	1 (8)	2 (8)	4 (21)	*0.242*
Decreased marginal zone-like B cells, *n* (%)	5 (9)	0 (0)	2 (8)	3 (16)	*0.157*

^$^ Two missing data

^*^ Some patients were documented for multiple bacteria

^**^ Rheumatoid arthritis (*n* = 2), ankylosing spondylitis (*n* = 2), polymyalgia rheumatica (*n* = 1), chronic inflammatory rheumatism (*n* = 1)

^***^ Type III cryoglobulinemia (*n* = 2), positive rheumatoid factor (*n* = 1), positive antinuclear antibodies > 1/1280 (*n* = 1)

**Fig. 1:**
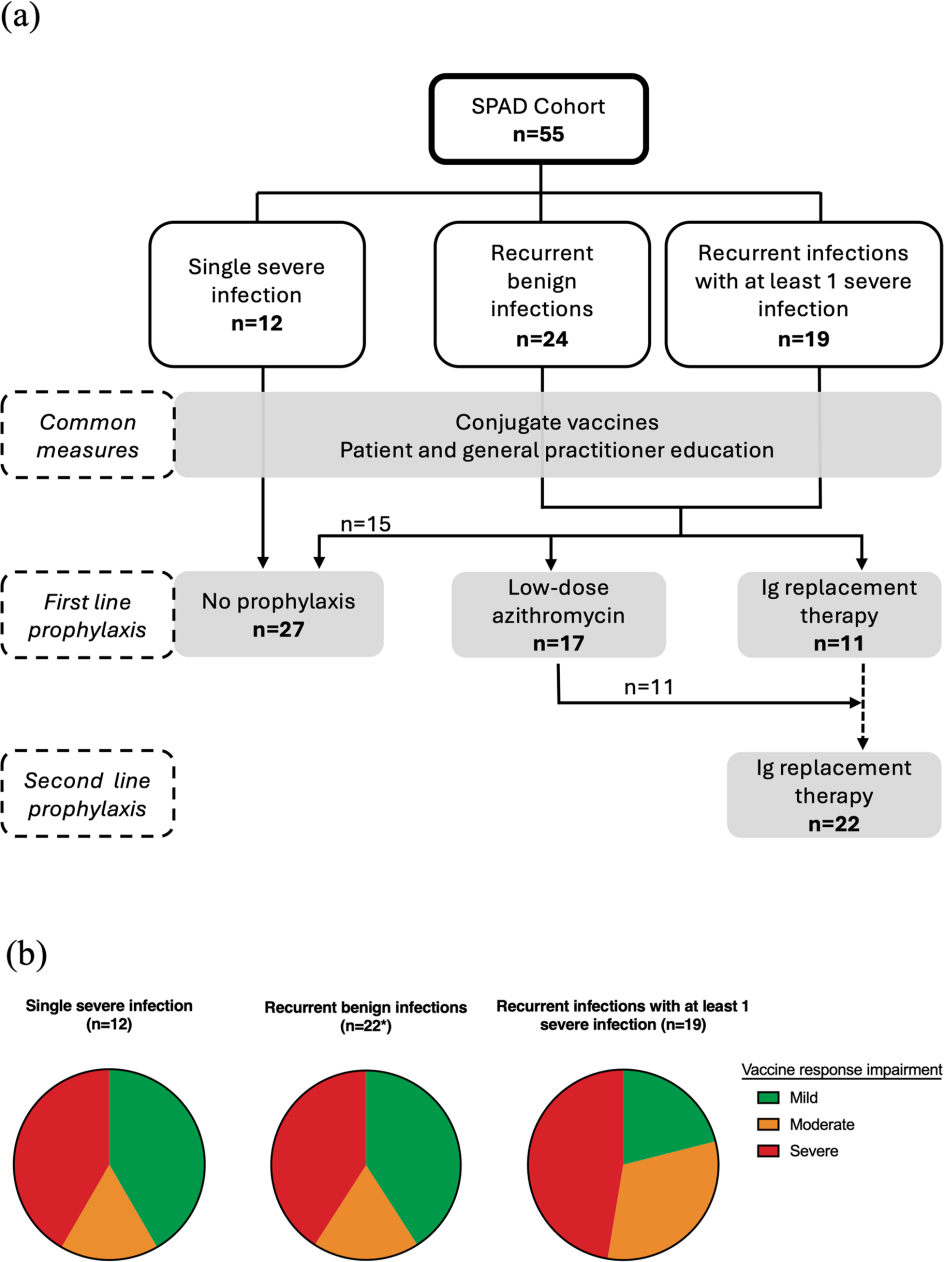
Infectious phenotypes of SPAD and patient management. **a** Distribution of SPAD phenotype among 3 patient groups: (i) a single severe infection, (ii) recurrent benign infections only and (iii) recurrent infections with at least 1 severe infection requiring hospitalization, with therapeutic management (**b**) Repartition of biologic phenotypes vaccine response impairment among the 3 infectious groups, defined as mild (green), moderate (orange) or severe (red) vaccine response impairment (according to [7]) * 2 missing data

**Fig. 2:**
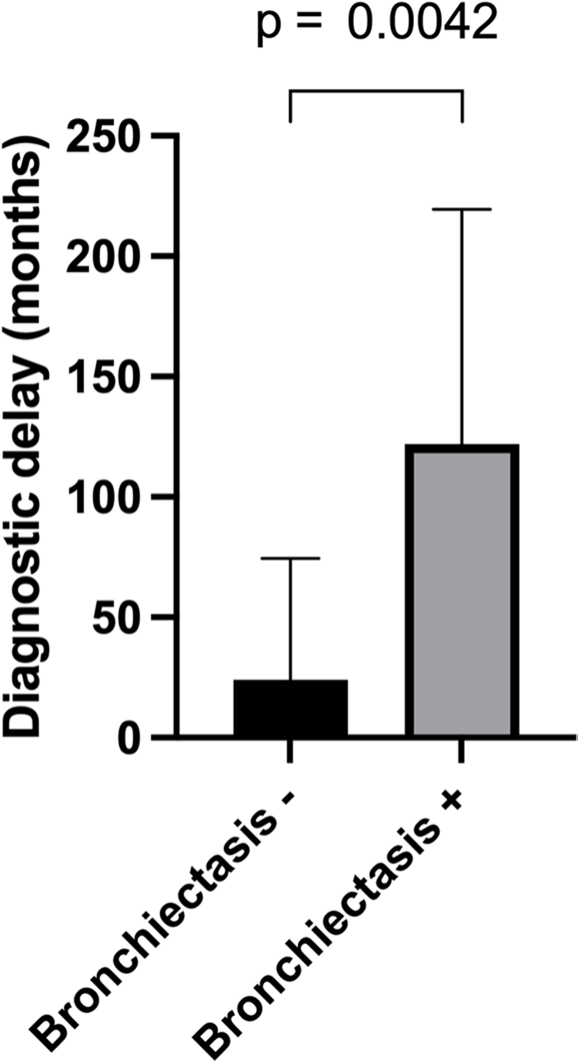
Diagnostic delay according to presence or absence of bronchiectasis

Infectious manifestations at diagnosis were previously reported in 47 patients [8, 11]. Three groups were identified according to the infectious phenotype: (i) 12 (22%) patients presented with a single severe infection (meningitis [*n* = 8], acute respiratory distress syndrome due to pneumonia [*n* = 2], pneumococcal endophthalmitis [*n* = 1], pneumococcal spondylodiscitis [*n* = 1]), (ii) 24 (44%) patients presented with recurrent benign infections, and (iii) 19 (34%) presented with recurrent infections with at least one severe infection requiring hospitalization (*n* = 8) or recurrent severe infections (*n* = 11) (Fig. 1a, Table 1). The degree of vaccine response impairment did not differ between the various groups identified based on infectious clinical presentation (Fig. 1b). Thirteen patients (23%) presented with bronchiectasis (diffuse [*n* = 7], localized [*n* = 6]). Patients with recurrent infections were diagnosed after a median delay of 74.5 (33–167) months. Diagnostic delay was significantly higher in patients presenting with bronchiectasis at diagnosis (24 months [14.5–74.5] versus 122 months [33–219.5], *p* = 0.0042) (Fig. 2). T-cell, B-cell, and NK-cell counts were available in 48 patients: T- and B-cell counts were normal in all patients, but a decreased NK-cell count (below 100/mm^3^) was present in 12 cases (25%). Naive/memory B-cell counts were available in 30 patients and 7 had decreased relative counts of (Table 1, see also Figure SI1).

### Non-infectious Characteristics At Diagnosis

Allergic and/or inflammatory disorders were present in 21 patients (38%), comprising asthma (*n* = 12), rheumatic diseases (*n* = 6; including rheumatoid arthritis [RA] [*n* = 2], ankylosing spondylitis [*n* = 2], polymyalgia rheumatica, and unclassified chronic inflammatory rheumatism [*n* = 1]), autoimmune thyroiditis (*n* = 4), Sjögren’s syndromes (*n* = 2), cutaneous psoriasis (*n* = 2), and a recently described auto-inflammatory disorder known as systemic inflammatory trunk recurrent acute macular eruption (SITRAME syndrome, *n* = 1) [13]. These patients with allergic and/or inflammatory disorders were not undergoing treatment with systemic steroids or immunosuppressants at the time of anti-PS response assessment, but 9 patients would have required oral steroids and/or immunosuppressants for conditions such as rheumatic diseases, cutaneous psoriasis, and connective tissue diseases. In the two cases of RA, the use of steroids and immunosuppressants was contraindicated due to recurrent benign and/or severe infections, resulting in progressive joints destruction and a decline in quality of life prior to the diagnosis of SPAD (Table 1). No history of lymphadenopathy, splenomegaly, or neoplasia was documented in our cohort.

### Follow-up

The median duration of follow-up was 67.8 months (48–84 months). No deaths occurred during the study period.

All patients were educated about warning signs necessitating prompt consultation with their general practitioner. All patients received conjugate vaccines against *Streptococcus pneumoniae* (PCV13) and *Haemophilus infuenzae* type b (HibCV), and against *Neisseria meningitidis* in case of previous meningococcal infection. Pre- and post-vaccination paired serological assessments were only available for 5 patients receiving PCV13 and 5 patients receiving HibCV: low response were observed in 4 and 2 cases, respectively (Figure SI2). Some patients kept low serum antibody levels despite repeated conjugate vaccines (data not shown).

Seventeen patients underwent treatment with low-dose azithromycin as “bronchial anti-inflammatory therapy” (250 or 500 mg three times a week). The efficacy of this treatment was deemed in 7 out of 15 evaluated (47%, with 5 partial and 2 complete improvements; 2 losses to follow-up prior to evaluation). After azithromycin failure or partial improvement, 11 patients subsequently received IgRT, including one patient whose bronchiectasis progressed despite azithromycin (Fig. 1a).

Overall, 22 patients were treated with IgRT at an initial dose of 0.1 g/kg/week (subcutaneous route) or 0.4 g/kg/month (intravenous route). This choice was made by the referent specialist in case of recurrent infections, with 3 additional criteria: a history of severe infection (*n* = 7; *e.g.* requiring ICU hospitalizations – Figure SI3), the presence of bronchiectasis (*n* = 8) and/or highly frequent RTIs (*n* = 8 patients without severe infections or bronchiectasis, were prescribed between 6 to 12 antibiotics per year).

**Fig. 3:**
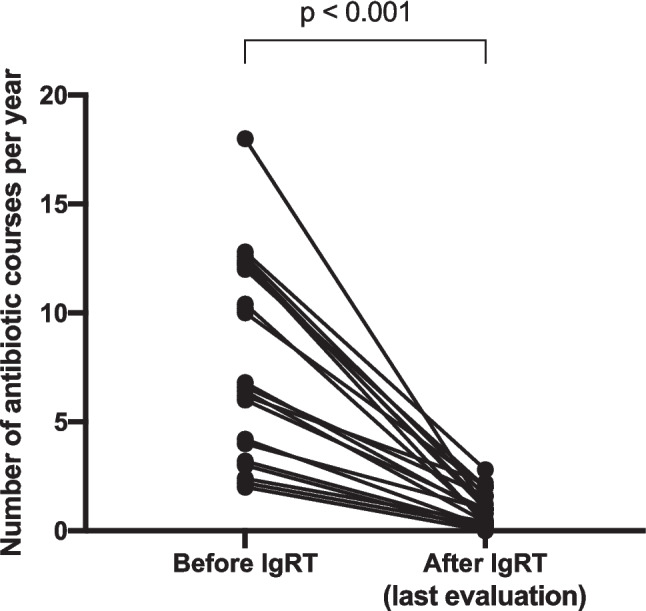
Change in the number of antibiotic courses per year before and after introducing IgRT (*n* = 22)

The mean frequency of antibiotic courses decreased significantly from 7.9 (2–18) courses per year to 0.7 (0–2) course per year following IgRT (*p* < 0.001), with a median follow-up period of 46 months (27–73.3 months) (Fig. 3). Mild vaccine response impairment was present in groups of patients with and without bronchiectasis, with and without IgRT (respectively 25% and 36%; 25% and 39%—Figure SI4). One patient with severe chronic obstructive pulmonary disease (COPD) and bronchiectasis, requiring supplemental oxygen therapy and non-invasive ventilation, had a poor response to both PCV13 and PPV23. He received azithromycin and IgRT was then added: bacterial infections decreased from 1–2 per month prior to diagnosis to 3–4 per year, and lung transplantation, which was contraindicated, was reconsidered.

Among the 12 patients who presented exclusively with a single severe infection, no recurrence of a relevant bacterial infection was observed during a median follow-up of 85 months (80.5–104.5 months) after diagnosis, despite absence of any prophylaxis excepted conjugate vaccines.

One patient with Sjögren’s syndrome later developed systemic lupus erythematosus and thrombotic thrombocytopenic purpura. Despite various immunosuppressant therapies (including corticoids and rituximab), no infections occurred while IgRT was maintained. Patients with RA who received IgRT for their specific anti-polysaccharide antibody deficiency were also optimally managed for their inflammatory disorder with agents such as rituximab, abatacept, tocilizumab, and certolizumab, without experiencing new infections (Figure SI5).

**Fig. 4:**
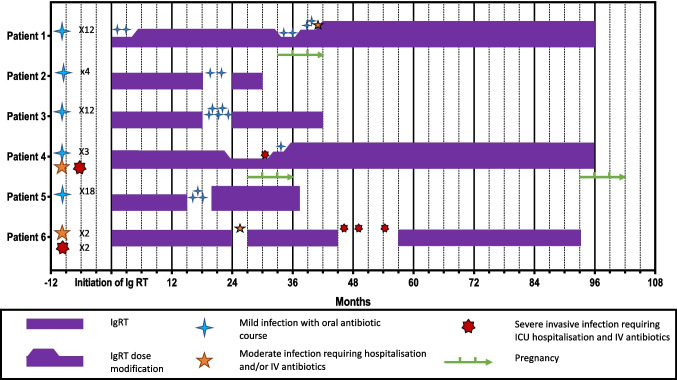
Infectious events in 6 patients with reduction or discontinuation of IgRT. ICU, intensive care unit; IgRT, immunoglobulin replacement therapy; IV, intravenous

No significant decrease was observed in IgA and IgM levels 47 patients with available follow-up data, no significant decrease in IgG levels was observed in 26 patients not receiving IgRT. IgRT was discontinued by 4 patients; however, infections recurred in all cases, and IgRT was started again. In 2 pregnant women who previously experienced a dose reduction of IgRT, new bacterial infections occurred, and the dose was subsequently increased, with a recovered efficacy in preventing infections (Fig. 4).

## Discussion

We present a cohort of 55 adult patients with SPAD, detailing their characteristics at diagnosis and their outcomes after a median follow-up period exceeding 5 years.

We observed a notable prevalence of allergic and inflammatory diseases among patients with SPAD. Twenty-two percent (*n* = 12/55) were diagnosed with asthma, occasionally accompanied by severe and recurrent asthma exacerbations linked to bacterial infections, with 10 patients experiencing recurrent bacterial sinusitis. In a large cohort of asthmatic patients, Lee et al. reported a prevalence of antibody deficiencies in 5% of patients [14]. This is of importance as U/LRTIs can precipitate asthma exacerbations [14, 15]. The frequency of antibody deficiency in severe chronic rhinosinusitis (CRS) patients, with or without asthma, is also high, with up to 40% of SPAD [16–18]. We also report on a severe COPD patient with monthly bacterial infections despite smoking cessation. In a case series of 42 COPD patients with very frequent exacerbations of infectious origin, 29 were diagnosed with PID (8 common variable immunodeficiency [CVID], 20 SPAD, and 1 IgA deficiency), and 22 patients benefited from IgRT (less exacerbations, reduction of steroids and antibiotics use) [19]. As the diagnosis of SPAD may represent a significant turning point in their management, assessing the anti-PS response can be crucial in patients with severe asthma, CRS and COPD, and recurrent RTIs [20–22].

Thirteen patients in our cohort were presented with autoimmune and/or inflammatory disorders. Only few studies have been conducted on vaccine response to polysaccharides, mostly in RA patients, and using only the OVA [23, 24]. Our case series suggest that SPAD diagnosis must be considered in these patients in case of recurrent bacterial infections. Indeed, 9 of our patients would have required oral steroids and/or immunosuppressants for their autoimmune/allergic conditions. However, these patients received suboptimal treatment due to their recurrent infections. Following the diagnosis of SPAD, IgRT prevented recurrent severe RTI and facilitated the introduction of appropriate immunosuppressants.

The criteria defining a good or poor anti-PS response were established based on an expert consensus in 2012 and grounded in various pediatric and adult studies [2, 7] using a complex and non-automated SSA [10]. We also proposed that, notwithstanding its limitations, the OVA could be utilized when a post-vaccination antibody titer below 110 mg/L was recorded, as this consistently correlated with a poor PPV23 response using the SSA [11]. Six patients in our cohort were diagnosed using this method, and five of them received IgRT, which effectively prevented recurrent infections. It is important to note that a defective response may be observed in healthy individuals, depending on the choice of specific antibody levels, of pre/post immunization fold-increases, of the number of tested serotypes, and of the methodology employed [17, 25]. This is why it is important to consider this hypothesis in adult patients with unexplained bacterial infections [8]. In the present cohort, we identified 3 infectious phenotypes: (i) a single unexplained severe infection, (ii) recurrent benign infections, and (iii) recurrent benign and ≥ 1 severe infection.

Of the 12 patients in our study who presented with a single severe infection, none experienced a recurrence of bacterial infections during a median follow-up of 85 months (80.5–104.5 months) after diagnosis. This raises questions regarding the utility of investigating SPAD after a single severe infection, particularly considering the prevalence of low anti-PS responses in healthy individuals [17]. Pre/post conjugate vaccine immunization assessments (PCV13 and HibCV) were available in only five patients. Despite the lack of standardization, it seems that conjugate vaccines induce a poor immune response in some SPAD patients. Further studies are needed to determine the response to conjugate vaccines according to the SSA and their clinical benefit in preventing recurrent RTIs and severe infections in these patients.

Patients with antibody deficiency experiencing recurrent infections often face delayed diagnosis and may develop bronchiectasis. In a study conducted by Van Kessel and colleagues in 2005, 26 patients with bronchiectasis of unknown etiology were examined, revealing that 15 of them were non-responders to PPV23 [26]. Early diagnosis is crucial for averting life-threatening infections and lung disease, and the high incidence of bronchiectasis in our cohort of SPAD patients underscores the consequences of delayed diagnosis.

Therapeutic management remains non-standardized and largely relies on expert recommendations and clinician preferences. This management typically includes administration of conjugate vaccines, patient education regarding the risks of bacterial infections, and, in cases of persistent recurrent infections, initiation of preventive antibiotic therapy or IgRT [27, 28]. In our cohort, all patients received conjugate vaccines and were educated on symptoms and situations warranting prompt medical attention and early initiation of antibiotic therapy. A letter was also sent to their primary care physicians for the same purpose.

Azithromycin was found to be effective in reducing the incidence of infections, decreasing the risk of hospitalization, and enhancing quality of life in patients with CVID and X-linked agammaglobulinemia [29] Among the 17 SPAD patients initially treated with azithromycin in our cohort, 11 eventually required a switch to IgRT due to poor effectiveness of azithromycin. The decision to initiate IgRT was based on the severity and the frequency of infections, and on the presence of bronchiectasis. IgRT demonstrated efficacy in 100% of cases, whether employed as first- or second-line therapy. This observed efficacy surpassed that reported in previous case series [16–18, 30, 31]. This difference can be attributed to the systematic exclusion of all potential differential diagnoses prior to SPAD diagnosis in our cohort, as well as the higher frequency of infections, and the bacterial infections requiring antibiotics (and not mild infections, or likely viral infections) [30]. This efficacy was also highlighted by the recurrence of bacterial infections, sometimes severe, in patients who discontinued IgRT or reduced the dose. Monitoring of immunoglobulin levels in our adult patients receiving IgRT did not indicate a progression to a quantitative deficit such as CVID, as it can encountered in children [32].

Finally, we also wondered if the phenotype of anti-PS responsiveness was associated with prognosis [7, 25, 33]. This classification suggested by experts has never been compared to the clinical phenotype of patients. If we consider the ‘mild’, ‘moderate’ or ‘severe’ phenotypes, we do not see any clear association between the level of anti-PS response and the prognosis, since at least 25% of patients with recurrent severe infections, bronchiectasis or IgRT initiation, can have a so-called “mild” phenotype. This raises questions about the relevance of phenotype responsiveness, as the infectious complications may better define the severity of SPAD and its management.

## Conclusion

**Fig. 5:**
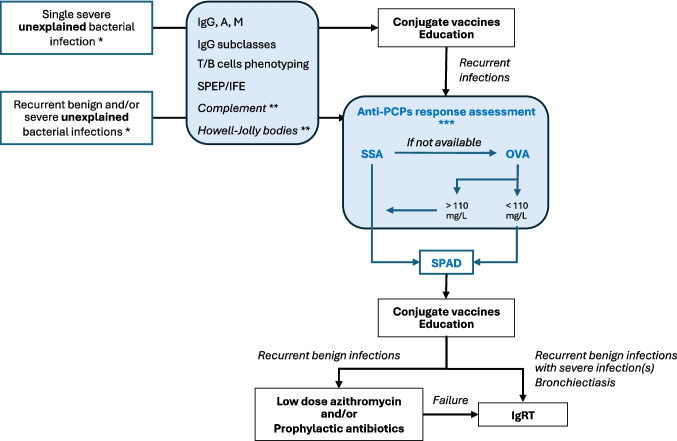
SPAD diagnosis and management according to the infectious complications. *Relevant bacterial infections are RTIs and/or invasive infection with encapsulated bacteria, when the patient’s history or investigations do not explain the frequency or the severity of the infections. **Complement explorations and search of Howell-Jolly bodies are indicated in invasive infection with encapsulated bacteria. *** SSA must be used if locally available, OVA can be used in other cases, but SSA will be mandatory in many cases (see Lopez et al. [11]). IgRT: immunoglobulin replacement therapy; IFE, immunofixation electrophoresis; OVA, overall anti-pneumococcal response assay; RTIs: respiratory tract infections; SPAD: specific antibody antibody deficiency; SPEP: serum protein electrophoresis; SSA: single-serotype assay

In summary, our study addresses the diagnostic challenges and management strategies associated with SPAD in a cohort of 55 adult patients. This diagnosis should also be considered in patients exhibiting autoimmune manifestations alongside such infectious complications, or in those with severe chronic lung disease experiencing difficult-to-treat exacerbations and/or unexplained bronchiectasis. Patients who had a single unexplained bacterial infection and did not have any other infections after diagnosis should be tested for other immunodeficiencies and receive conjugate vaccines but may not be tested for SPAD (Fig. 5). In patients with recurrent bacterial RTIs, SPAD diagnosis must be considered in absence of any other immunodeficiency. IgRT must be considered as a first-line treatment, or after low dose azithromycin or prophylactic antibiotics, in case of frequent recurrent RTIs, history of severe infections or bronchiectasis, (Fig. 5). Prospective studies are warranted to confirm the benefit of this therapeutic strategy.

## Supplementary Information

Below is the link to the electronic supplementary material.

Supplementary file1 (DOCX 14947 KB)

## Data Availability

No datasets were generated or analysed during the current study.

## Abbreviations

PID: Primary immunodeficiency
U/LRTI: Upper/lower respiratory tract infection
SPAD: Specific anti-polysaccharide antibody deficiency
PS: Polysaccharide
PnPS: Pneumococcal capsular polysaccharide
AAAAI: American Academy of Allergy, Asthma & Immunology
IgRT: Immunoglobulin replacement therapy
PPV23: 23-Valent pneumococcal polysaccharide vaccine
PCV13: 13-Valent pneumococcal conjugate vaccine
CVID: Common variable immunodeficiency
CRS: Chronic rhinosinusitis
RA: Rheumatoid arthritis
COPD: Chronic obstructive pulmonary disease
HibCV: *Haemophilus influenzae* Serotype B conjugate vaccine
OVA: Overall anti-pneumococcal response assay
SSA: Single serotype assay
SITRAM: Systemic inflammatory trunk recurrent acute macular eruption
IQR: Interquartile range
