# Supplementary material for: Diagnosis, Characteristics, and Outcome of Selective Anti-polysaccharide Antibody Deficiencies In A Retrospective Cohort of 55 Adult Patients
Source: J Clin Immunol. 2025 Mar 17;45(1):82. doi: 10.1007/s10875-025-01874-2 (PMC11914230; doi:10.1007/s10875-025-01874-2)
Supplement: Supplementary file 1 — Supplementary file1 (DOCX 14947 KB) [file 10875_2025_1874_MOESM1_ESM.docx]

**Supplementary Information**

FIGURE SI1. Decreased relative counts of switched memory B cells (CD27+IgD-IgM-, n = 7) and marginal zone-like B cells (CD27+IgD+IgM+, n = 5) in SPAD patients.

(a) (b)

FIGURE SI2. Assessment of response to conjugate vaccines in SPAD patients. (a) OVA for anti-pneumococcal antibodies before and after vaccination with PCV13. (b) Assessment of anti-*Haemophilus infuenzae* serotype B antibodies before and after vaccination with HibCV.

FIGURE SI3. A case of a SPAD patient with progressive worsening of bronchiectasis.

1: On-flu secondary infection by *Streptococcus pyogenes*, with refractory ARDS requiring v-vECLS and bacteriema, multiple thrombosis including spleen ischemia, necrosis of the extremities requiring amputation of 4 limbs; 2,4,5,7: Pneumonia (*Haemophilus influenzae*); 3: Pneumonia (no documentation); 6: Bronchitis (*Moraxella catarrhalis*). ARDS, acute respiratory distress syndrome; v-vECLS, veno-venous extracorporeal life support; ICU, intensive care unit; IV, intravenous; IgRT, immunoglobulin replacement therapy

FIGURE SI4. Repartition of vaccine response impairment among patients with/without bronchiectasis and with/without IgR

^*^ 2 missing data;

^$^ 1 missing data

FIGURE SI5. A case of a SPAD patient with rheumatoid arthritis.

1: Pneumonia with empyema (*Streptococcus pneumoniae*); 2, 3, 4, 8: Pneumonia - ICU with septic shock (*Klebsiella pneumoniae*, ESBL); 5: Bilateral maxillary sinusitis (*Klebsiella pneumoniae*, ESBL); 6, 7: Pneumonia (no documentation); 9, 10: Bilateral pneumonia - ICU with respiratory failure (No documentation). ICU, Intensive care unit; ESBL, extended spectrum beta-lactamase; RA, rheumatoid arthritis; IV, intravenous
